# Systematic analysis of programmed cell death–related genes in sepsis reveals immune heterogeneity and enables patient stratification

**DOI:** 10.3389/fcell.2026.1805864

**Published:** 2026-05-18

**Authors:** Guangliang Zhang, Kai Dai, Qingyu Meng, Tianzhen Hua, Yi Yao, Hong Zhu, Wei Liu

**Affiliations:** 1 Department of Burns and Plastic Surgery, The Fourth Medical Center of Chinese PLA General Hospital, Beijing, China; 2 Chinese PLA Medical School, Beijing, China; 3 Department of Critical Care Medicine, The Fourth Medical Center of the Chinese PLA General Hospital, Beijing, China; 4 Department of General Surgery, The First Medical Center, PLA General Hospital, Beijing, China; 5 Department of Critical Care Medicine, Central Hospital of Dalian University of Technology, Dalian, Liaoning, China

**Keywords:** immune heterogeneity, programmed cell death, sepsis, single-cell RNA sequencing, stratification

## Abstract

**Background:**

Sepsis is a life-threatening syndrome characterized by dysregulated host response to infection. Programmed cell death (PCD) pathways, including apoptosis, necroptosis, and pyroptosis, play a critical role in sepsis pathophysiology. However, the crosstalk between PCD, metabolic reprogramming, and immune heterogeneity remains poorly understood.

**Methods:**

We analyzed transcriptomic data from the GSE57065 and GSE95233 datasets. Differentially expressed genes (DEGs) were intersected with programmed cell death-related genes (PCDRGs). Consensus clustering was performed to identify molecular subtypes. A risk stratification model was constructed using LASSO and Random Forest algorithms. The immune landscape was deconvoluted using CIBERSORT. To validate cellular mechanisms, we integrated single-cell RNA sequencing (scRNA-seq) data, analyzing cell type-specific expression, intercellular communication (CellChat), and pathway enrichment. Additionally, the reliability of the identified signature was verified in a murine sepsis model (burn injury combined with *Pseudomonas aeruginosa* infection) using quantitative real-time PCR (qPCR).

**Results:**

We identified 14 dysregulated PCDRGs in sepsis. Clustering based on these genes revealed two distinct patient subgroups: an “adaptive-dominant” cluster (enriched in T cell activation) and an “innate-dominant” cluster (enriched in neutrophil degranulation and heme metabolism). A 5-gene risk signature (IFNGR1, PYGL, JAK2, HMGB2, BMX) was constructed, which robustly stratified patients in the discovery and external validation cohorts (AUC > 0.97). Experimental validation in septic mice confirmed that the expression levels of all five hub genes were significantly upregulated in peripheral blood, consistent with the human transcriptomic findings. High-risk patients exhibited severe immune imbalance characterized by neutrophil/monocyte expansion and lymphocyte depletion. Single-cell analysis confirmed that hub genes were predominantly expressed in myeloid lineages. Furthermore, high-risk patients displayed intensified Monocyte-Dendritic Cell (DC) crosstalk driven by inflammatory signaling, with transcriptional programs in these cells converging on neutrophil degranulation pathways.

**Conclusion:**

This study establishes a robust PCDRG-based risk signature that captures the immune–metabolic heterogeneity of sepsis. The signature enables patient stratification and reflects disease-associated immune states. Our findings suggest a potential association between dysregulated PCD genes in myeloid cells and an innate immune–dominant, hyper-inflammatory phenotype, providing potential biomarkers and therapeutic insights for sepsis.

## 1 Introduction

Sepsis remains a leading cause of mortality in Intensive Care Units (ICUs) globally, representing a complex physiological response to infection that leads to multi-organ dysfunction (Singer et al., 2016). The pathophysiology of sepsis is biphasic and heterogeneous, involving an initial hyper-inflammatory “cytokine storm” often followed by a prolonged state of (Hotchkiss et al., 2013). Despite advances in supportive care, specific therapeutic interventions have largely failed (Cohen, 2002), primarily due to the lack of tools to stratify patients based on their underlying molecular heterogeneity (Rudd et al., 2020; Marshall, 2014; Rhodes et al., 2017).

Programmed cell death (PCD)—encompassing apoptosis, necroptosis, and pyroptosis—is a fundamental mechanism regulating immune homeostasis (Bedoui et al., 2020). While various forms of PCD, such as ferroptosis and cuproptosis, have recently emerged, we focused on these three classical pathways due to their established roles as primary drivers of sepsis pathology. Specifically, apoptosis is a major contributor to sepsis-induced lymphopenia and subsequent immunoparalysis, while the lytic nature of pyroptosis and necroptosis facilitates the massive release of pro-inflammatory cytokines, fueling the “cytokine storm” (Delano and Ward, 2016; Jorgensen et al., 2017; Shi et al., 2017). Recent evidence suggests that these pathways do not operate in isolation but interact through complex “PANoptosis” networks, where they are co-regulated by the PANoptosome complex (Karki and Kanneganti, 2021; Malireddi et al., 2019). Therefore, analyzing the crosstalk between these three core PCD forms provides a comprehensive perspective on the immune-metabolic dysregulation in sepsis (van der Pol et al., 2017). Furthermore, immune cell function is intimately tied to metabolic states; for instance, the shift from oxidative phosphorylation to glycolysis is linked to pro-inflammatory phenotypes in myeloid cells (O’Neill et al., 2016; Cheng et al., 2014).

While bulk transcriptomics has identified general sepsis signatures, the specific contribution of PCD-related genes (PCDRGs) to the diverse immune and metabolic landscapes of sepsis patients is not fully characterized (Davenport et al., 2016; Sweeney et al., 2018). Moreover, resolving these signatures at the single-cell level is crucial to understanding which cell types are associated with disease heterogeneity and severity (Reyes et al., 2020).

**FIGURE 1:**
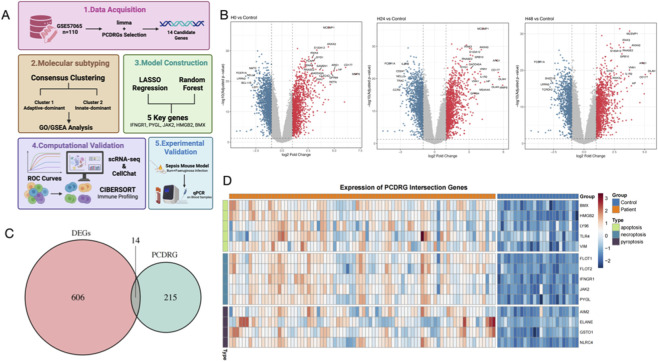
**(A)** Workflow of this study, including dataset selection, DEG/PCDRG intersection, clustering, model construction, validation, single-cell analysis, and mouse validation. **(B)** Volcano plots illustrate widespread transcriptional alterations in septic patients compared to healthy controls at 0, 24, and 48 h post-onset (GSE57065; n = 28 healthy controls and n = 82 sepsis samples). **(C)** Intersection of differentially expressed genes with a curated PCDRG panel identified 14 overlapping genes. **(D)** Heatmap visualization reveals distinct expression patterns of these 14 PCDRGs. Notably, key regulators of apoptosis, necroptosis, and pyroptosis (e.g., HMGB2, AIM2, NLRC4, JAK2) were significantly upregulated in sepsis, highlighting the concurrent activation of multiple cell death pathways during disease progression.

In this study, we hypothesized that the expression patterns of PCDRGs could stratify sepsis patients into distinct immune-metabolic subgroups. We integrated large-scale bulk transcriptomic cohorts with single-cell RNA sequencing (scRNA-seq) data to construct a robust risk stratification model and elucidate the cellular crosstalk driving sepsis severity (Figure 1A).

## 2 Materials and methods

### 2.1 Data acquisition and processing

Publicly available microarray datasets (GSE57065 and GSE95233) were obtained from the Gene Expression Omnibus (GEO). GSE57065 served as the discovery cohort, containing peripheral blood samples from septic patients at 0 h, 24 h, and 48 h, and healthy controls (Davenport et al., 2016). GSE95233 was used as an independent external validation cohort (Scicluna et al., 2017). Single-cell RNA-seq datasets of peripheral blood mononuclear cells (PBMCs) from septic patients and controls were acquired for validation (Reyes et al., 2020).

### 2.2 Identification of differentially expressed PCDRGs

Differential expression analysis was performed using the limma package in R (Ritchie et al., 2015). Genes with an adjusted *p*-value < 0.05 and |log2 Fold Change| > 1 were considered significant. A curated list of Programmed Cell Death-Related Genes (PCDRGs) was intersected with the DEGs to identify sepsis-associated PCDRGs. Genes were further cross-referenced with KEGG and GO cell death–related pathways (Kanehisa and Goto, 2000).

### 2.3 Unsupervised clustering and functional enrichment

Consensus clustering was applied to stratify patients based on the expression of intersected PCDRGs. Gene Ontology (GO) and Kyoto Encyclopedia of Genes and Genomes (KEGG) enrichment analyses were performed on cluster-specific marker genes using clusterProfiler (Wilkerson and Hayes, 2010). Gene Set Enrichment Analysis (GSEA) was utilized to identify activated biological pathways between clusters (Subramanian et al., 2005).

### 2.4 Construction of the risk stratification model

We employed machine learning algorithms to develop a PCDRG-based risk stratification model. Before model construction, gene expression data were log2-transformed and normalized. Candidate genes were first subjected to least absolute shrinkage and selection operator (LASSO) regression using the “glmnet” R package. A 10-fold cross-validation was performed to determine the optimal penalty parameter (λ), and genes with non-zero coefficients were retained (Friedman et al., 2010; Breiman, 2001).

Subsequently, a Random Forest model (ntree = 500) was applied to further evaluate feature importance and refine the candidate gene set. The final risk score was calculated as a linear combination of gene expression values weighted by their corresponding LASSO coefficients.

Patients were stratified into high- and low-risk groups based on the median risk score. The predictive performance of the model was evaluated using receiver operating characteristic (ROC) curve analysis.

### 2.5 Immune infiltration analysis

The CIBERSORT algorithm was used to quantify the relative proportions of 22 infiltrating immune cell types in bulk transcriptomic samples (Newman et al., 2015). We compared immune cell composition between high- and low-risk groups and analyzed correlations between hub gene expression and immune cell abundance.

### 2.6 Single-cell RNA-seq analysis

scRNA-seq data were processed using the Seurat package (Hao et al., 2021). Quality control, normalization, and dimensionality reduction (PCA, UMAP) were performed. Cell types were annotated based on canonical markers (e.g., CD3D for T cells, CD14 for Monocytes, CD79A for B cells). The “Risk Score” for single cells was inferred based on the aggregate expression of the hub genes.

### 2.7 Cell-cell communication analysis

Intercellular communication networks were inferred using the CellChat R package (Jin et al., 2021). We compared the number and strength of ligand-receptor interactions between high-risk and low-risk groups and identified signaling pathways significantly enriched in the high-risk group.

### 2.8 Murine sepsis model and qPCR validation

#### 2.8.1 Animal model establishment

Male C57BL/6 mice (8–10 weeks old, 20–25 g) were purchased and housed under specific pathogen-free conditions with a 12-h light/dark cycle. The study was approved by the Institutional Animal Care and Use Committee (IACUC). To establish the sepsis model, a “two-hit” protocol involving thermal injury and bacterial infection was employed. Mice were anesthetized with isoflurane, and the dorsal hair was shaved (Stieritz et al., 1975). A full-thickness scald burn covering 30% of the total body surface area (TBSA) was induced by immersing the dorsal skin in 90 °C water for 10 s. Immediately post-burn, mice were resuscitated with 1 mL of sterile saline intraperitoneally (Stieritz et al., 1975). Subsequently, 1 × 10^5^ CFU of *Pseudomonas aeruginosa* (strain PAO1) was injected subcutaneously at the burn site to induce sepsis. The control group (Sham) underwent anesthesia and shaving but was immersed in lukewarm water (37 °C) and received saline injection without bacteria.

#### 2.8.2 RNA extraction and qPCR

Peripheral blood samples were collected 24 h post-injury. Total RNA was extracted using the TRIzol reagent (Invitrogen, CA, United States) according to the manufacturer’s protocol. RNA concentration and purity were assessed using a NanoDrop spectrophotometer. Complementary DNA (cDNA) was synthesized from 1 μg of total RNA using the PrimeScript™ RT Reagent Kit (Takara, Japan). Quantitative Real-Time PCR (qPCR) was performed using TB Green® Premix Ex Taq™ II (Takara) on a LightCycler® 480 System (Roche). The reaction conditions were as follows: 95 °C for 30 s, followed by 40 cycles of 95 °C for 5 s and 60 °C for 30 s. The relative expression levels of the target genes were calculated using the 2^−ΔΔCt^ method, with *Gapdh* serving as the endogenous control.

#### 2.8.3 Primer sequences

The specific primer sequences for the mouse genes used in this study are listed below (Spandidos et al., 2010):

Ifngr1

Forward: 5′- CTT​GAA​CCC​TGT​CGT​ATG​CTG​G -3′

Reverse: 5′- TTG​GTG​CAG​GAA​TCA​GTC​CAG​G -3′

Pygl

Forward: 5′- GGC​AGA​AGT​GGT​GAA​CAA​TGA​CC -3′

Reverse: 5′- TCC​GAT​AGG​TCT​GTG​GCT​GGA​A -3′

Jak2

Forward: 5′- GCT​ACC​AGA​TGG​AAA​CTG​TGC​G -3′

Reverse: 5′- GCC​TCT​GTA​ATG​TTG​GTG​AGA​TC -3′

Hmgb2

Forward: 5′- GAT​GTG​GTC​TGA​GCA​ATC​TGC​C -3′

Reverse: 5′- CCT​GCT​TCA​CTT​TTG​CCC​TTG​G -3′

Bmx

Forward: 5′- TCA​ACC​AAG​GCC​AAC​AAG​GT -3′

Reverse: 5′- CCT​CTG​GAG​CTG​ACC​ACT​TG -3′

Gapdh (Internal Control)

Forward: 5′- AGG​TCG​GTG​TGA​ACG​GAT​TTG -3′

Reverse: 5′- TGT​AGA​CCA​TGT​AGT​TGA​GGT​CA -3′

### 2.9 Statistical analysis

All statistical analyses were performed using R software (version 4.1.2). The “glmnet” package (v4.1) was used for LASSO regression, and the “randomForest” package (v4.7) was used for feature importance ranking.” Continuous variables were compared using the Wilcoxon rank-sum test or Student’s t-test, as appropriate. Receiver operating characteristic (ROC) curve analysis was performed to assess diagnostic performance, and the area under the curve (AUC) was calculated. To control the false discovery rate (FDR) in high-throughput analyses, multiple-testing corrections were applied using the Benjamini–Hochberg (BH) method. Specifically, BH correction was employed for: (i) differential expression analysis via the limma and Seurat packages; (ii) functional enrichment analyses (GO/KEGG) via the clusterProfiler package; and (iii) intercellular communication probability estimation in CellChat. For correlation matrices, p-values were adjusted using the BH method to ensure the robustness of gene-cell associations. A two-sided *p*-value < 0.05 was considered statistically significant.

## 3 Result

### 3.1 Differentially expressed genes intersect with programmed cell death–related genes in sepsis

To systematically explore the involvement of programmed cell death (PCD) in sepsis progression, we first analyzed the transcriptomic dynamics of the GSE57065 dataset. We compared peripheral blood gene expression between septic patients and healthy controls at three critical time points (0 h, 24 h, and 48 h post-onset). Volcano plots revealed extensive transcriptional reprogramming, with thousands of genes showing significant upregulation or downregulation at all time points (Figure 1B), reflecting the intense host response to infection.

By intersecting these differentially expressed genes (DEGs) with a curated panel of Programmed Cell Death-Related Genes (PCDRGs), we identified 14 overlapping genes that were consistently dysregulated (Figure 1C). Heatmap visualization further elucidated the expression landscape of these 14 genes. A distinct separation was observed between septic patients and healthy controls. Notably, key regulators of diverse cell death pathways—including pyroptosis (*AIM2, NLRC4*), necroptosis (*HMGB2, TLR4*), and apoptosis (*JAK2*)—were markedly upregulated in septic samples across all time points (Figure 1D) (Place and Kanneganti, 2018; Linkermann et al., 2014).

### 3.2 PCDRG-based clustering stratifies sepsis patients into distinct immune–metabolic subgroups

**FIGURE 2:**
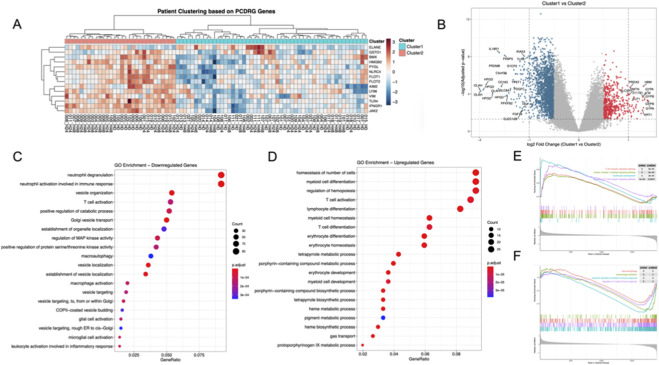
**(A)** Consensus clustering stratified sepsis patients into two stable molecular subtypes (Cluster 1 and Cluster 2) based on the 14 PCDRGs. **(B)** Volcano plot displays divergent gene expression profiles between the two clusters. **(C,D)** Functional enrichment analysis shows Cluster 1 is associated with T cell activation and vesicle organization **(C)**, while Cluster 2 is enriched in neutrophil degranulation and heme metabolic processes **(D)**. **(E,F)** GSEA confirms that Cluster 1 is dominant in adaptive immune signaling, whereas Cluster 2 exhibits enrichment in innate immune responses and macrophage activation.

We hypothesized that the expression heterogeneity of these 14 PCDRGs could reflect underlying biological subtypes of sepsis. Unsupervised consensus clustering identified two stable subgroups of patients, designated as Cluster 1 and Cluster 2 (Figure 2A).

Differential expression analysis between the two clusters revealed a large number of DEGs, indicating significant molecular divergence (Figure 2B). Functional enrichment analysis of downregulated genes demonstrated significant enrichment in neutrophil degranulation, neutrophil activation, vesicle organization, and T cell activation pathways (Figure 2C). Conversely, upregulated genes were strongly associated with erythroid and myeloid differentiation, hematopoietic regulation, and porphyrin/heme metabolic processes (Leliefeld et al., 2016) (Figure 2D).

Gene Set Enrichment Analysis (GSEA) further supported these findings. Cluster 1 was enriched in pathways related to T cell receptor signaling and adaptive immune activation (Venet and Monneret, 2018) (Figure 2E), whereas Cluster 2 showed enrichment in macrophage activation, neutrophil-mediated immune responses, and inflammatory signaling pathways (Figure 2F). Together, these results indicate that PCDRG-based clustering captures immune–metabolic heterogeneity in sepsis, separating patients into an adaptive immunity–associated group and an innate immunity–dominant group (Manz and Boettcher, 2014).

### 3.3 Construction of a PCDRG-based risk score model to stratify sepsis patients

**FIGURE 3:**
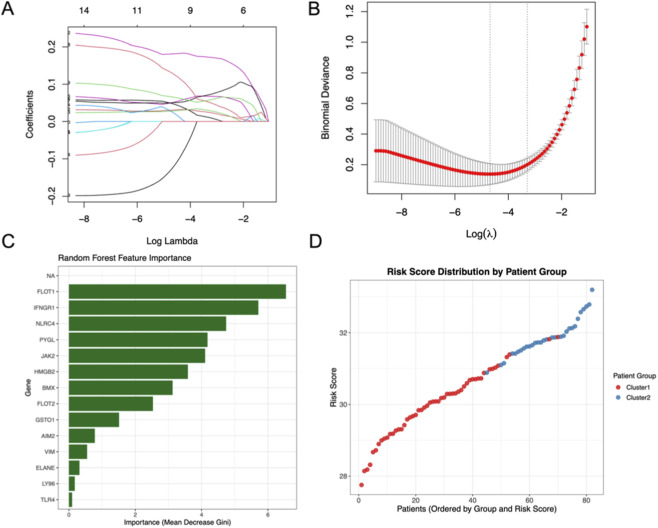
**(A,B)** LASSO regression analysis selected optimal features for patient stratification, with the penalty parameter determined by minimum binomial deviance (n = 110 total samples). **(C)** Random Forest feature ranking identified top predictors contributing to cluster discrimination, including FLOT1, IFNGR1, and JAK2. **(D)** A risk score was generated using a 5-gene signature (IFNGR1, PYGL, JAK2, HMGB2, BMX). The distribution plot demonstrates clear separation, with Cluster 2 (innate-dominant) patients exhibiting significantly higher risk scores, validating the model’s efficacy in capturing sepsis heterogeneity.

To further establish a prognostic system based on programmed cell death–related genes (PCDRGs), we applied machine learning methods to the intersected candidate genes. Using LASSO regression, a subset of features was retained as optimal predictors for patient clustering, with the penalty parameter (λ) determined by minimum binomial deviance (Figures 3A,B).

To validate gene importance, a random forest classifier was constructed. The feature ranking confirmed several genes—including FLOT1, IFNGR1, NLRC4, PYGL, and JAK2—as the top contributors to cluster discrimination (Figure 3C).

We then generated a PCDRG-based risk score using the weighted expression of the selected hub genes. The distribution of risk scores demonstrated clear separation between Cluster 1 and Cluster 2 patients, with Cluster 2 showing significantly higher risk scores (Figure 3D). These results indicate that the constructed immune–metabolic risk scoring system effectively stratifies sepsis patients and reflects underlying programmed cell death–related molecular heterogeneity.

### 3.4 External validation confirms strong diagnostic performance of PCDRG-derived hub genes

**FIGURE 4:**
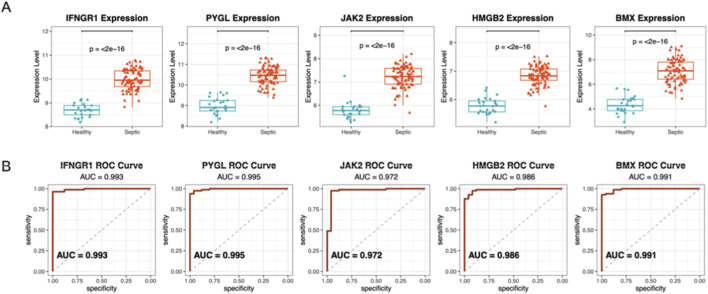
**(A)** Expression analysis in an independent external cohort (GSE95233; n = 22 healthy controls and n = 51 sepsis patients) confirms that all five hub genes (IFNGR1, PYGL, JAK2, HMGB2, BMX) are significantly upregulated in septic patients compared to healthy controls (p < 2e-16). **(B)** ROC curve analysis assesses the diagnostic accuracy of the signature. All five genes demonstrated exceptional discriminatory power with AUC values > 0.97, confirming their robustness as stable biomarkers for sepsis diagnosis across independent patient populations.

To ensure the robustness of our 5-gene signature, we validated it in an independent external dataset (GSE95233). Consistent with the discovery cohort, expression analysis showed that all five hub genes (*IFNGR1, PYGL, JAK2, HMGB2, BMX*) were significantly upregulated in septic patients compared to healthy controls (Figure 4A, *p* < 2e-16).

We evaluated the diagnostic accuracy using Receiver Operating Characteristic (ROC) curves. The model demonstrated exceptional discriminative power, with Area Under the Curve (AUC) values exceeding 0.97 for all five genes: IFNGR1 (AUC = 0.993), PYGL (AUC = 0.995), JAK2 (AUC = 0.972), HMGB2 (AUC = 0.986), and BMX (AUC = 0.991) (Figure 4B). These results strongly support the stability and potential clinical utility of this PCDRG-based signature for sepsis diagnosis and stratification.

### 3.5 Immune infiltration analysis reveals neutrophil enrichment and T cell suppression in high-risk patients

**FIGURE 5:**
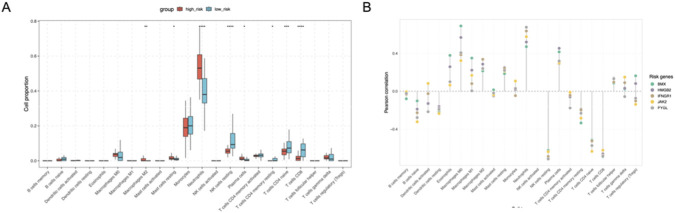
**(A)** CIBERSORT analysis (n = 110 samples) reveals significant differences in immune composition. High-risk patients exhibit markedly expanded neutrophils and monocytes but depleted CD4+/CD8+ T cells and NK cells, indicative of an immunosuppressive phenotype. **(B)** Correlation analysis links hub gene expression to cell types. All five genes showed strong positive correlations with innate effectors (neutrophils/monocytes) and negative correlations with adaptive lymphocytes, suggesting the risk signature reflects an innate immunity driven biological state.

We next investigated the cellular landscape associated with the high-risk phenotype using CIBERSORT deconvolution. A dramatic shift in immune composition was inferred using CIBERSORT deconvolution. High-risk patients were characterized by a massive expansion of innate effector cells, specifically neutrophils and monocytes, while simultaneously showing a profound depletion of adaptive lymphocytes, including CD4^+^ T cells, CD8^+^ T cells, and NK cells (Figure 5A). This profile matches the clinical picture of severe sepsis-induced immunosuppression (or immunoparalysis) (Boomer et al., 2014). High-risk patients exhibited markedly increased proportions of neutrophils and monocytes, accompanied by reduced frequencies of NK cells and multiple T cell subsets, including CD4^+^ and CD8^+^ T cells. These results suggest a trend where high-risk patients are characterized by an estimated neutrophil-dominant, lymphocyte-suppressed immune phenotype.

We next assessed the association between the expression of hub genes and immune cell composition. Correlation analysis revealed that all five hub genes (BMX, HMGB2, IFNGR1, JAK2, and PYGL) were strongly and positively correlated with neutrophils and monocytes, while displaying negative correlations with NK cells and T cell subset (Figure 5B). These findings further support the notion that the PCDRG-derived risk signature reflects an innate immunity–driven immunological state in sepsis, which may underlie the poor prognosis of high-risk patients.

### 3.6 Single-cell comparison of high- and low-risk groups highlights innate–adaptive immune imbalance and hub gene expression patterns

**FIGURE 6:**
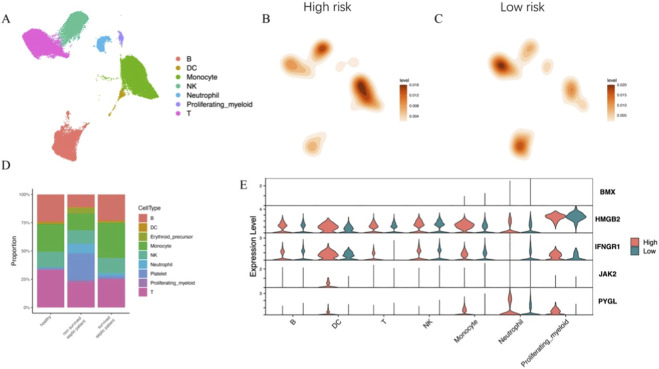
**(A–C)** UMAP density plots project patient risk groups onto single-cell clusters. High-risk patients show dense accumulation in Monocyte and Neutrophil clusters **(B)**, whereas low-risk patients retain higher density in T cell clusters **(C)**. **(D)** Comparative analysis of cell composition reveals that non-surviving septic patients are characterized by a marked increase in neutrophil and monocyte proportions coupled with a reduction in T and NK cells, validating the immune imbalance patterns observed in the bulk transcriptomic high-risk group. **(E)** Violin plots demonstrate that the five hub genes (BMX, HMGB2, IFNGR1, JAK2, PYGL) are predominantly expressed in monocytes, neutrophils, and proliferating myeloid cells, confirming that the prognostic risk signature is biologically driven by myeloid cell dysregulation.

To validate the immune–metabolic heterogeneity observed in bulk transcriptomic analyses, we integrated published single-cell RNA-seq datasets of peripheral blood from septic patients and healthy controls (Villani et al., 2017). After quality control, normalization, and dimensionality reduction, major immune cell subsets were clearly separated by UMAP visualization (Figure 6A). UMAP visualization confirmed clear separation of major immune cell populations, including B cells, DCs, T cells, NK cells, monocytes, neutrophils, and proliferating myeloid cells (Figure 6A). Density plots revealed that high-risk patients were enriched in monocytes and neutrophils, whereas low-risk patients showed higher frequencies of T cells (Figures 6B,C). This shift reflects an innate immunity–dominated phenotype in high-risk patients, consistent with the immune infiltration patterns observed in bulk transcriptomic analyses.

Comparative analysis of cell composition across clinical groups revealed distinct immune cell distributions (Figure 6D). Non-surviving septic patients displayed a marked increase in neutrophil and monocyte proportions, coupled with decreased frequencies of T cells and NK cells. In contrast, surviving patients retained relatively balanced immune cell profiles. We next explored the cell type–specific expression of the five hub genes. Violin plots demonstrated that BMX, HMGB2, IFNGR1, JAK2, and PYGL were predominantly expressed in monocytes, neutrophils, and proliferating myeloid cells, while showing limited expression in lymphoid lineages (Figure 6E). These results suggest that the risk-associated genes are primarily expressed in innate immune cells, further linking programmed cell death–related metabolic dysregulation to neutrophil and monocyte hyperactivation in high-risk sepsis.

### 3.7 Cell–cell communication analysis reveals intensified monocyte–DC interactions in high-risk patients

**FIGURE 7:**
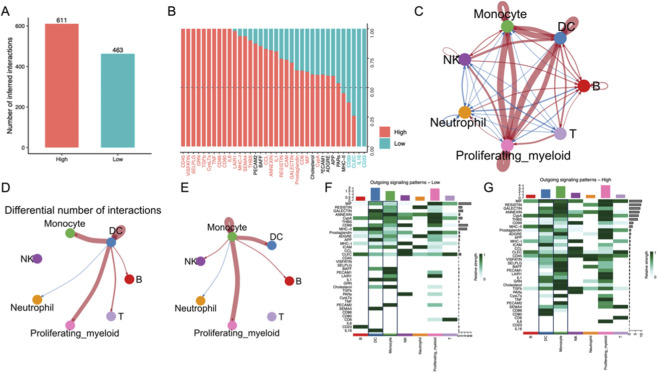
**(A)** CellChat analysis indicates a higher frequency of cell-cell interactions in the high-risk group compared to low-risk (611 vs. 463). **(B)** Information flow analysis shows enrichment of pro-inflammatory pathways in high-risk patients. **(C–E)** Network visualization identifies Monocytes and DCs as central communication hubs. **(F,G)** Outgoing signaling patterns reveal that Monocytes and DCs in high-risk patients are potentially linked to the inflammatory amplification loop specifically through enhanced cytokine and costimulatory signaling molecules.

To further investigate intercellular communication underlying the immune heterogeneity of sepsis, we applied CellChat to compare ligand–receptor signaling between high- and low-risk groups. High-risk patients were predicted to exhibit a markedly higher number of estimated cell–cell interactions compared with low-risk patients (611 vs. 463; Figure 7A). Relative information flow analysis revealed that several inflammatory and costimulatory signaling pathways were predominantly enriched in the high-risk group, whereas homeostatic signals were more active in the low-risk group (Tang et al., 2021; Roger et al., 2001) (Figure 7B).

Network visualization demonstrated a denser intercellular communication network in the high-risk group, with monocytes and dendritic cells (DCs) serving as central hubs (Figure 7C). Differential interaction mapping highlighted a significant increase in monocyte–DC crosstalk in the high-risk group (Figures 7D,E). Outgoing signaling patterns further confirmed stronger monocyte- and DC-derived signals in high-risk patients, particularly in pro-inflammatory pathways (Figures 7F,G).

These findings suggest that heightened intercellular communication, especially enhanced monocyte–DC interactions, potentially contributes to the innate immune dominance observed in our model and inflammatory amplification characteristic of high-risk sepsis patients.

### 3.8 Upregulated genes in monocytes and DCs from high-risk patients are enriched in neutrophil degranulation pathways

**FIGURE 8:**
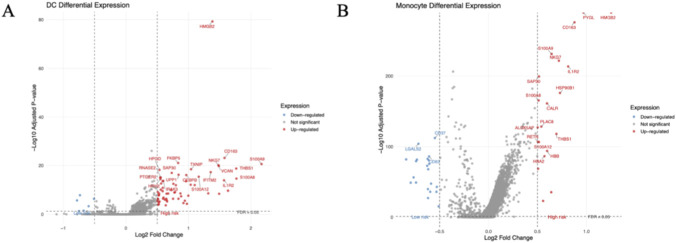
**(A,B)** Differential expression analysis reveals extensive gene upregulation in DCs **(A)** and Monocytes **(B)** from high-risk patients, including hub genes like HMGB2.

To further clarify the cellular mechanisms underlying the risk score–defined subgroups, we examined transcriptional changes in dendritic cells (DCs) and monocytes between high- and low-risk patients at the single-cell level. Differential expression analysis revealed extensive gene upregulation in high-risk DCs (Figure 8A) and monocytes (Figure 8B). Notably, hub genes such as HMGB2, PYGL, and IFNGR1 were among those significantly elevated in the high-risk group. Functional enrichment analysis demonstrated that upregulated genes in high-risk DCs were predominantly associated with neutrophil degranulation, macrophage activation, phagocytosis, and pro-inflammatory cytokine production. Similarly, upregulated genes in high-risk monocytes were enriched in granulocyte chemotaxis, antigen processing, and stress response pathways.

These results indicate that both DCs and monocytes in high-risk patients adopt a pro-inflammatory transcriptional program that converges on neutrophil-related effector functions (Silvestre-Roig et al., 2016). This provides mechanistic evidence that programmed cell death–related gene dysregulation is potentially linked to innate immune amplification through enhanced neutrophil degranulation and inflammatory signaling.

### 3.9 *In vivo* experimental validation confirms upregulation of hub genes in a murine sepsis model

**FIGURE 9:**
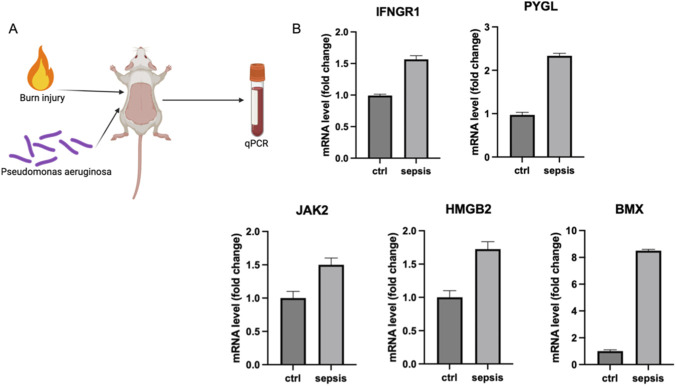
**(A)** Schematic diagram of the *in vivo* experimental design. A murine sepsis model was established by inducing burn injury followed by *Pseudomonas aeruginosa* infection to mimic clinical septic complications. Peripheral blood was collected for gene expression analysis. **(B)** Bar graphs showing the relative mRNA expression levels of the five hub genes (IFNGR1, PYGL, JAK2, HMGB2, and BMX) in the control (Ctrl) and sepsis groups as measured by qPCR. Consistent with the bioinformatic results, all five genes were significantly upregulated in the sepsis group (n = 6 mice per group). Data are presented as mean ± SD (Standard Deviation).

To further verify the biological reliability of the identified PCDRG signature *in vivo*, we established a clinically relevant murine sepsis model. Given that burn patients are highly susceptible to sepsis, we utilized a “two-hit” model involving burn injury followed by *Pseudomonas aeruginosa* infection to mimic severe septic conditions (Figure 9A). Peripheral blood samples were collected from the sepsis group and control group for transcriptional validation.

Quantitative Real-Time PCR (qPCR) analysis was performed to measure the expression levels of the five prognostic hub genes. Consistent with our bioinformatic discovery in human cohorts, the results demonstrated that the mRNA expression levels of IFNGR1, PYGL, JAK2, HMGB2, and BMX were all significantly upregulated in the septic mice compared to the healthy controls (Figure 9B). Notably, *BMX* exhibited the most dramatic elevation (over 8-fold), aligning with its role in stress responses and ischemia, while *HMGB2* and *PYGL* also showed robust upregulation (>1.5-fold and >2-fold, respectively). These experimental findings provide strong translational evidence that the 5-gene PCDRG signature is a stable and conserved feature of sepsis pathophysiology across species.

## 4 Discussion

Sepsis is a highly heterogeneous syndrome in which mortality often arises not solely from uncontrolled infection, but from the host’s failure to appropriately balance pathogen clearance with tissue tolerance. Excessive immune activation can be as deleterious as immune paralysis, underscoring the need for molecular frameworks that capture this dynamic imbalance. In this study, we constructed a robust programmed cell death–related gene (PCDRG)–based risk signature that not only stratifies sepsis patients based on molecular and immune characteristics, but also delineates the underlying immune–metabolic architecture associated with disease severity.

Unsupervised clustering based on PCDRG expression stratified sepsis patients into two dominant phenotypes. The “innate-dominant” cluster (Cluster 2) is characterized by myeloid expansion and lymphocyte suppression, a transcriptomic profile that mirrors the clinical hallmark of immunoparalysis (Hotchkiss et al., 2013; Delano and Ward, 2016). This state of protracted immune dysfunction resembles the previously described SRS1 endotype, which is associated with poor outcomes and secondary infections (Davenport et al., 2016). Conversely, the “adaptive-dominant” cluster (Cluster 1) aligns with the SRS2 endotype, maintaining relatively preserved T-cell programs and a more balanced immune response (Davenport et al., 2016).

This stratification offers a roadmap for precision therapy. Cluster 2 patients, being in a hyper-inflammatory state, are optimal candidates for anti-inflammatory interventions such as cytokine blockers or JAK inhibitors (e.g., Baricitinib), as identified by our drug-gene interaction analysis (Kalil et al., 2020). In contrast, Cluster 1 patients may benefit from immune-stimulatory agents, such as recombinant IL-7 or PD-1/PD-L1 inhibitors, if they transition into immune exhaustion (Hotchkiss et al., 2013; Venet and Monneret, 2018). Such a targeted approach could mitigate the “one-size-fits-all” failure observed in historical sepsis trials. Such a stratified approach could potentially improve the success rate of clinical trials by targeting the right treatment to the right patient (O’Sullivan et al., 2007).

The identified hub genes provide mechanistic insight into these divergent phenotypes. Beyond their individual predictive power, these five genes exhibit significant biological synergy. JAK2 and IFNGR1 constitute a central signaling axis for interferon-γ–mediated inflammatory amplification, while HMGB2 acts as a critical DAMP that triggers pyroptosis and NETosis (Oh et al., 2025). Furthermore, PYGL-mediated glycogenolysis provides the metabolic fuel for these innate responses (O’Sullivan et al., 2007; Chen et al., 2019). IFNGR1 and JAK2 constitute a direct signaling axis for interferon-gamma (O’Sullivan et al., 2007), while HMGB2 (Deng et al., 2022) (an alarmin) and PYGL (Cheng et al., 2014) (a metabolic enzyme) provide the inflammatory stimuli and metabolic energy required to sustain this pathway. Collectively, this integrated network orchestrates the hyper-inflammatory response in the innate-dominant subgroup, highlighting that our risk model captures a functional regulatory module rather than isolated markers.

Our single-cell analysis demonstrates that the risk signature is intrinsic to the myeloid compartment. High-risk patients exhibited expansion of neutrophils and monocytes with elevated expression of hub genes, whereas lymphoid populations were relatively depleted. Importantly, cell–cell communication analysis revealed intensified monocyte–dendritic cell (DC) crosstalk mediated by CD40 and MIF signaling axes in high-risk patients (Tang et al., 2021; Calandra et al., 2000). Macrophage Migration Inhibitory Factor (MIF) is a potent pro-inflammatory cytokine known to delay neutrophil apoptosis, prolong neutrophil survival, and amplify cytokine production (Roger et al., 2001; Pasparakis and Vandenabeele, 2015). This mechanism may contribute to neutrophil accumulation and excessive degranulation, a pathway we found to be strongly enriched in high-risk myeloid cells, thereby suggesting a potential link between PCDRG dysregulation and tissue injury.

Importantly, the exceptional performance of this 5-gene signature (AUC > 0.97) underscores its potential for clinical translation. The biological robustness of our 5-gene signature was further corroborated by *in vivo* experiments. Detection via qPCR could provide rapid results within hours, while ELISA and flow cytometry offer practical protein-level quantification for markers such as HMGB2 and IFNGR1. These targeted assays are significantly more cost-effective and accessible than high-throughput sequencing, making them ideal for rapid point-of-care (POC) risk stratification in intensive care settings (Sweeney et al., 2018).

Despite the robust performance of our risk signature, several limitations should be acknowledged. First, this study relied on retrospective datasets from public repositories. Although externally validated, the signature’s predictive power requires confirmation in large-scale, prospective clinical cohorts with well-annotated metadata. Second, the intrinsic heterogeneity of sepsis—including variations in infection source, pathogen type, and patient comorbidities—could not be fully controlled due to the limited clinical information available in public cohorts. Third, the immune landscape and intercellular communications were inferred using computational algorithms (CIBERSORT and CellChat). While these provide valuable insights, they represent estimations rather than definitive biological measurements and should ideally be cross-validated using flow cytometry or protein-level assays. Finally, although we validated the consistent upregulation of hub genes in a murine sepsis model, these findings primarily support gene-expression consistency across species. Future loss-of-function and gain-of-function studies are necessary to establish the direct causal mechanisms by which these PCDRGs drive immune dysfunction and tissue injury in sepsis. In addition, further studies incorporating long-term survival and multi-organ histopathology are warranted to fully elucidate the predictive value of this scoring system *in vivo*.

## 5 Conclusion

Our study provides a comprehensive framework linking programmed cell death–related transcriptional programs to immune–metabolic heterogeneity in sepsis. Through a combination of multi-cohort transcriptomic analysis and *in vivo* experimental validation,we identified a 5-gene PCDRG signature (*IFNGR1, PYGL, JAK2, HMGB2, BMX*) that is associated with sepsis severity. By integrating bulk and single-cell analyses with cell–cell communication inference, we highlight myeloid-driven inflammation and defective immune resolution as central features of high-risk sepsis. These findings offer potential biomarkers for patient stratification and suggest that targeting specific myeloid signaling axes (e.g., JAK2) may represent a potential strategy to modulate the lethal immunopathology of sepsis. Targeting dysregulated programmed cell death pathways may represent promising strategies for precision intervention in sepsis patients with adverse immune phenotypes.

## Data Availability

The original contributions presented in the study are included in the article/supplementary material, further inquiries can be directed to the corresponding authors.
